# Current Understanding of Guanylin Peptides Actions

**DOI:** 10.5402/2013/813648

**Published:** 2013-04-17

**Authors:** Aleksandra Sindic

**Affiliations:** Department of Physiology, School of Medicine, University of Zagreb, Salata 3, 10000 Zagreb, Croatia

## Abstract

Guanylin peptides (GPs) family includes guanylin (GN), uroguanylin (UGN), lymphoguanylin, and recently discovered renoguanylin. This growing family is proposed to be intestinal natriuretic peptides. After ingestion of a salty meal, GN and UGN are secreted into the intestinal lumen, where they inhibit sodium absorption and induce anion and water secretion. At the same conditions, those hormones stimulate renal electrolyte excretion by inducing natriuresis, kaliuresis, and diuresis and therefore prevent hypernatremia and hypervolemia after salty meals. In the intestine, a well-known receptor for GPs is guanylate cyclase C (GC-C) whose activation increases intracellular concentration of cGMP. However, in the kidney of GC-C-deficient mice, effects of GPs are unaltered, which could be by new cGMP-independent signaling pathway (G-protein-coupled receptor). This is not unusual as atrial natriuretic peptide also activates two different types of receptors: guanylate cylcase A and clearance receptor which is also G-protein coupled receptor. Physiological role of GPs in other organs (liver, pancreas, lung, sweat glands, and male reproductive system) needs to be discovered. However, it is known that they are involved in pathological conditions like cystic fibrosis, asthma, intestinal tumors, kidney and heart failure, obesity, and metabolic syndrome.

## 1. Introduction

The physiological importance of digestive system is not only to digest and absorb nutrients but also to prepare the body for an increase in blood concentrations of glucose, amino acids, electrolytes, and water. It is well known that several hormones secreted by intestine after a meal, like gastrin, secretin, cholecystokinin, and especially gastric inhibitory peptide increase insulin production even before the concentrations of glucose and amino acids significantly increase in the blood. Lennane et al. in 1975 showed that salt taken per os will increase secretion of electrolyte and water by the kidneys more than the same amount of the salt given intravenously, proposing the existence of intestinal hormones that regulate kidney function [1]. After a salty meal, intestinal natriuretic peptides also known as guanylin peptides (GPs) are secreted by intestine where the inhibit sodium absorption from intestinal lumen by inhibiting sodium/hydrogen exchange (NHE), increase bicarbonate and chloride secretion, inhibit water absorption [2, 3], and increase renal sodium and potassium secretion [4–6]. Effects of GPs on sodium transport in the intestine and the kidneys prevent postprandial hypernatremia, gastric inhibitory peptide has a major role in decreasing postprandial increase in plasmatic glucose concentration as well. The growing family of GPs has, up to date, four members: guanylin (GN), uroguanylin (UGN), lymphoguanylin, and renoguanylin. GN was the first GP isolated first from rat intestine [7]. A year later, the most abundant GP present in the urine, UGN was isolated from opossum urine [8]. The other two members of the GPs family: lymphoguanylin isolated from opossum lymphatic tissues [9] and renoguanylin found in eels [10]. Mammalian isoform of renoguanylin is not discovered yet. However, the last two members of guanylin peptide family are up to date less investigated and their physiological functions yet need to be discovered.

The importance of GPs in maintaining the homeostasis of water and electrolyte homeostasis working together with other natriuretic peptides (like atrial natriuretic peptide) and opposite to rennin-angiotensin-aldosterone system and arginine vasopressin seems to be very relevant since GPs appeared early in evolution as they are found in all examined animals (mammals, birds, and fishes).

## 2. Structure and Metabolism of Guanylin Peptides

**Figure 1:**
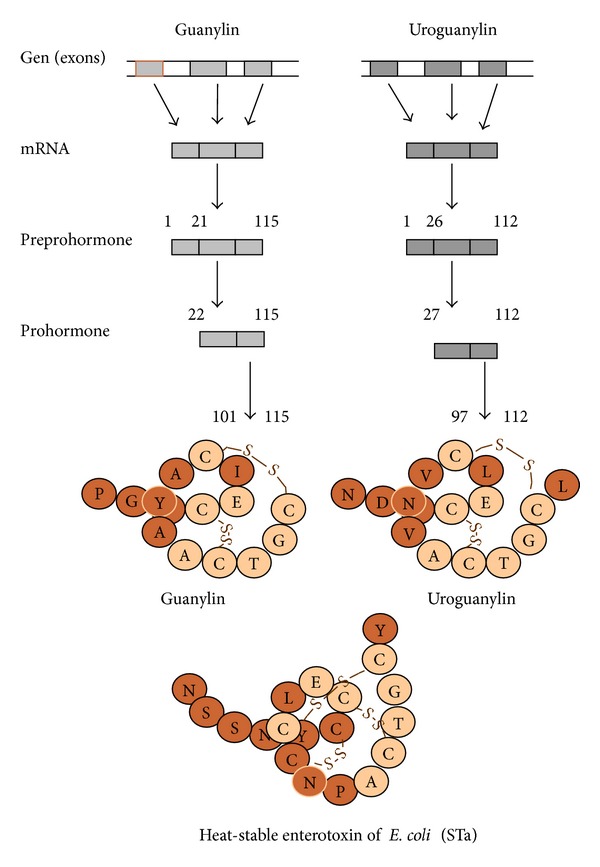
Structure and synthesis of human guanylin peptides. Guanylin and uroguanylin have 3 exons and 2 introns. Human preproguanylin has 115, proguanylin 94, and guanylin 15 amino acids. Human preprouroguanylin has 112, prouroguanylin 86, and uroguanylin 16 amino acids. Structures of human guanylin and uroguanylin are compared to the structure of heat-stable enterotoxin of *E. coli* (STa) at the bottom of the figure. Open circles show identical amino acids in both peptides. Solid circles represent amino acids responsible for peptide sensitivity to chymotrypsin.

Genes for natriuretic peptides secreted by the heart (atrial natriuretic peptide (ANP) and brain natriuretic peptide (BNP)) and by intestine (GN and UGN) are located at the first human chromosome and the fourth mouse chromosome [11]. Since GPs are by structure peptide hormones, they are synthesized as preprohormone, prohormone, and hormone (Figure 1). GN and UGN have 3 exons and 2 introns [12, 13]. Human pre-pro-GN has 115 amino acids. Is it cleaved to pro-GN which has 94 amino acids. The active hormone GN has 15 amino acids [14]. The production of human UGN is the same. Human pre pro-UGN has 112 amino acids and it is cleaved to pro-UGN with 86 amino acids. Active human UGN has 16 amino acids [15, 16]. However, it is suggested that the circulating form of UGN has 24 amino acids [17]. The difference in the function of those two types of UGN needs to be established.

GN and UGN have two disulfide bonds between cysteins which are essential for their activity (Figure 1) [14, 16, 22]. Today, we know that main receptor for GPs was first described as receptor for the heat-stable enterotoxin of *Escherichia coli* (STa) [23]. STa has 19 amino acids and three disulfide bonds which could be the reason for its stronger and unregulated activation on the GPs receptor. Stronger activation of GPs receptor by STa leads to more potent secretion of electrolyte and water into the intestinal lumen which caused secretory diarrhea [24, 25]. Since the discovery of receptors/binding sites for STa which is exogenous enterotoxin, in intestine as well as other extraintestinal tissue like kidneys, we started to look for endogenous ligands and first discovered GN and soon after UGN. 

Human GN is known as guanylate cyclase activating peptide-1; GCAP-1 consists of 15 amino acids and possesses two disulfide bonds between two cysteins (between 4–12 and 7–15 positions) (Figure 1) [14, 22]. From 15 amino acids, human GN shares similarity in 10 amino acids with GN from eel, opossum, rat, pig, and guinea pig with the highest similarity to pig GN with a difference only in 2 amino acids (Table 1). GN is present in the plasma mostly as its pro GN form in concentration of 30–40 pM [26, 27].

Human UGN is also known as guanylate cyclase activating peptide-2, GCAP-2. UGN has 16 amino acids and has two disulfide bonds between 4–12 and 7–15 positions similar to GN (Figure 1) [16]. Eel and pig isoforms of UGN also have 16 amino acids while opossum, rat, and guinea pig UGNs have 15 amino acids while they are missing the last amino acid. UGNs from all listed species (Table 1) share 9 amino acids at particular positions [28]. In both GN and UGN structures cysteins are preserved which are necessary for the development of disulfide bonds and activity of GPs. 

In contrast to GN, 60–90% of circulating UGN in the plasma is in the form of active hormone in a concentration of 5–7 pM [29, 30]. However, those findings are still in dispute. Recently, circulating plasma pro-UGN was suggested to mediate enterorenal signaling. It is suggested that filtrated pro-UGN is cut by proteases present in the tubular lumen to the active form of UGN [31, 32]. Structural differences between GN and UGN in different species are given in Table 1.

The structural difference between GN and UGN which lies in their sensitivity towards proteases is especially important for the physiological role of the GPs in the kidney. GN has a tyrosine as its ninth amino acid which makes it sensitive to proteases, mostly chymotrypsin. UGN has an asparagine as its ninth amino acid and is therefore protected form proteases present in tubular lumen. However, in the kidney filtrated pro-UGN is not protected and could be cut by tubular proteases [33, 34]. Therefore, GN is degraded by chymotrypsin present in different parts of the kidney nephrons [4, 35] and is consequently not present in urine.

After a salty meal, the intestine is producing GPs in the gut lumen (inhibition of the water and electrolyte absorption). At the same time, intestine produces GPs into the blood. It is proposed that GPs secreted form intestine induced natriuresis, kaliuresis, and diuresis in the kidney after glomerular filtration. Intestinal GPs when located in the tubular lumen act on receptors located at apical membrane of tubular cells. Even the proteins with ten times higher molecular weight, like myoglobin, are filtrated through glomerular membrane almost as easy as water (filterability is 75% of water), suggesting that even pro-GPs present in the plasma could be sufficiently filtrated and present in the lumen of different nephron segments. However, recent research suggests that in addition to endocrine function of the GPs secreted from the intestine, the other source of GPs and their precursors in tubular lumen could be released from the kidney cells. Kidney secretes GPs only locally into the tubular lumen where they act as paracrine peptides but not in the blood [20].

mRNA for GPs could be found in other organs as well, like adrenal glands, reproductive system, lung, and pancreas, where the importance of GPs secretion is, as far as we known, local regulation of membrane transport systems, and it is still not very well understood [36–38]. Although the mRNA for GPs is present in numerous tissues, the intestine is considered to be the only source for GPs present in the blood [29, 39, 40].

Both endocrine and paracrine secretion of the GPs via intestine are stimulated by parasympathetic system via activation of n.vagus [41, 42]. This vagal stimulation could have importance in UGN function in the stomach while UGN is produced by enterochromaffin-like cells where acetylcholine also regulates production of histamine which regulates HCl production of the stomach [43]. Interestingly, it is known that in patients with Zn^2+^ deficiency have secretory diarrhea. Cousins laboratory showed when Zn^2+^ deficiency is induced in rats, unregulated expression of UGN in enterocytes increased, which could explain secretory diarrhea in humans [44–47].

## 3. Signaling Pathways of Guanylin Peptides in the Intestine

Expression of GN and UGN in intestine is well regulated by salt ingestion. High-salt diet leads to the secretion of GN and UGN into the intestinal lumen. On the other hand, low salt intake decreases mRNA levels of GN and UGN in the rat digestive tract [3, 48], which is suggested to be a protective mechanism in how to spare sodium during periods of salt restriction in the food. 

**Figure 2:**
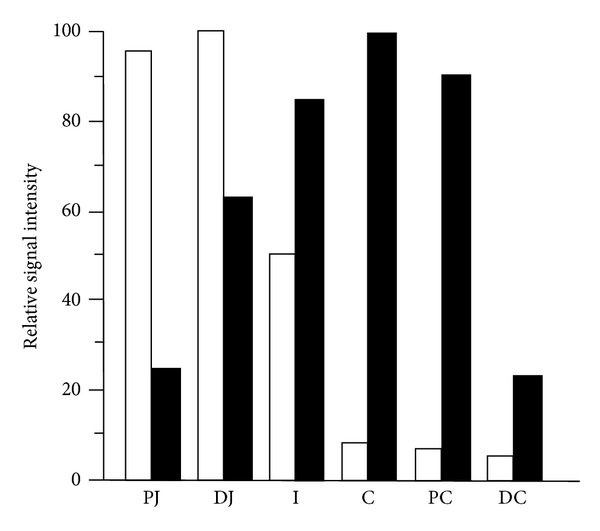
Distribution of mRNA for guanylin (black columns) and uroguanylin (white columns) in digestive system. Guanylin is present in all parts of gastrointestinal tract with highest expression in the distal parts of the tract. In contrast, uroguanylin is mostly present in proximal parts where it could play a role in regulating pH after emptying stomach content. PJ: proximal jejunum, DJ: distal jejunum, I: ileum, C: cecum, PC: proximal colon, DC: distal colon (modified from Whitaker et al. [18]).

GN is mainly present in goblet and epithelial cells of the colonic mucosa while UGN presents in enterochromaffin cells of the small intestine [2, 3, 49, 50]. mRNA for GN is located from duodenum to distal colon from lower to higher expression along the intestine (Figure 2) [51, 52]. UGN is present along the gastrointestinal tract with the highest expression in the duodenum [18, 38, 49, 51, 53, 54, 77].

**Figure 3:**
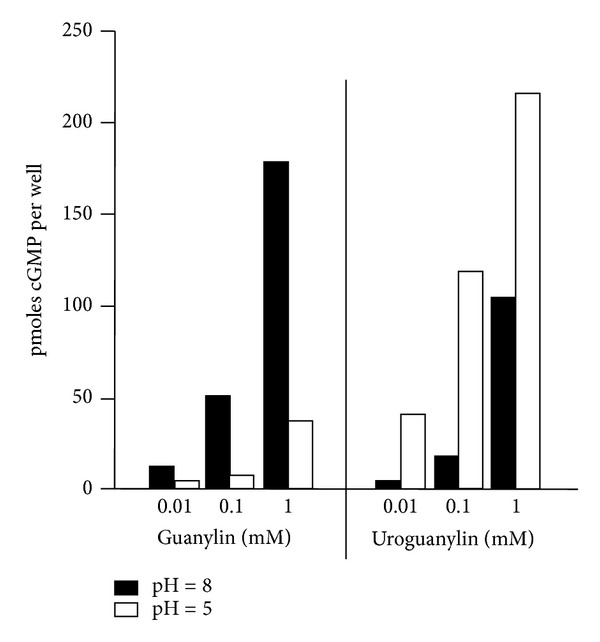
Guanylin (GN) and uroguanylin (UGN) increasing the intracellular concentration of cGMP are pH dependent. GN is more potent at pH 8.0, and UGN is more potent at pH 5.0 (modified from Hamra et al. [19]).

Proximal part of the digestive system, mainly duodenum, developed powerful mechanisms to protect epithelial cells against acid secretion from stomach. A well-know mechanism is inhibition of gastric emptying with intestinal hormones. Cholecystokinin is released from the jejunum mucosa as response to the presence of the fatty acids in intestinal lumen. Secretin is secreted in duodenum as a response to low pH duodenal content, and in addition to inhibition of stomach emptying it also induces pancreatic secretion rich in bicarbonate. UGN could play a significant role in epithelial protection by increasing pH of duodenal lumen via inhibition of hydrogen secretion (by NHE inhibition) and increase in bicarbonate secretion. The very important mechanism in those protective effects is pH sensitivity of GPs action. The effects of GN and UGN on concentration of intracellular cGMP after activation of their receptor guanylate cyclase C (GC-C) is pH dependent (Figure 3). The increase in intracellular concentration of cGMP after UGN stimulation is higher at pH 5 compared to pH 8. In opposition to that increase in cGMP, concentration induced by GN is higher at pH 8. Those findings correspond to the expression of the UGN in proximal part of digestive tract where pH is lower than in distal part where is more alkaline and GN is produced more, which potentiate effects of GPs via GC-C-, cGMP-dependent signaling pathway. Furthermore, pH sensitivity of UGN intracellular cGMP accumulation is also important in the duodenum. To neutralize the low pH after stomach empting in addition to pancreatic secretion, duodenum secretes bicarbonate. This secretion is cGMP dependent and could be a result of UGN action which is even more potentiated at low pH conditions. It was suggested by Hamra et al. (1997) that this pH dependence affects the ligand/receptor interaction depending on the N terminal ends of the UGN and GN molecules (Figure 1) [19].

**Figure 4:**
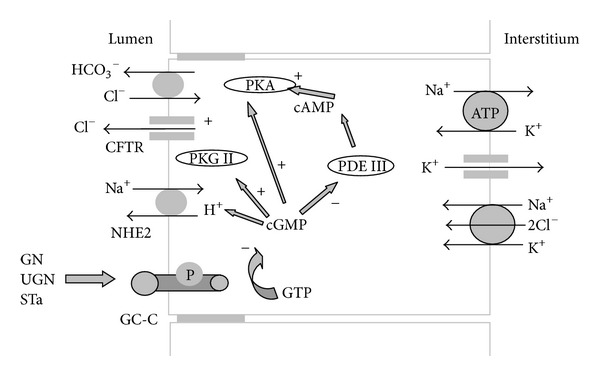
GC-C-dependent signaling pathway of guanylin peptides in the intestine. Guanylin (GN), uroguanylin (UGN), and heat-stable enterotoxin of *E. coli* (STa) activate GC-C which increases intracellular concentration of cGMP which in the enterocytes causes the following: inhibits the Na^+^/H^+^ exchanger type 2 (NHE2) and activates protein kinase G type II (PKG II), activates protein kinase A (PKA) directly or indirectly by inhibition of phosphodiesterase III (PDE III) followed by increase in intracellular cAMP concentration. PKA and PKG II activate Cl^−^ and HCO_3_^−^ secretion via activation of cystic fibrosis transmembrane regulator (CFTR) followed by an activation of the member of Slc26 family which exchanges bicarbonate for chloride.

As you can see at Figure 4. UGN increases bicarbonate secretions via CFTR, cGMP-dependent mechanism involving members of Slc26 transporter family. The Slc26 family has up to date 11 members. It exchanges Cl^−^ with sulfate, iodide, formate, oxalate, hydroxyl ion, and bicarbonate, whereas other function as Cl^−^ channels. Slc26a3 (DRA), Slc26a6 (PAT-1, CFEX), and Slc26a9 are involved in bicarbonate secretion in digestive tract and pancreas via CFTR-dependent pathway. Recently, Slc26a4 (pendrin) was suggested as target for UGN-dependent electrolyte transport in intercalated cells of kidney cortical collecting duct (see later) [55]. In the future, additional research should be done to investigate the possible effects of GPs on other members of Slc26 family in the way to determine existence of CFTR-independent signaling pathway for GPs. Furthermore, UGN inhibits H^+^ secretion via inhibition of sodium/proton exchanger (NHE) and therefore decreases H^+^ concentration in the duodenum [19]. As described above, this cGMP/GC-C-dependent UGN effects are more pronounced at acidic pH after gastric emptying, inducing stronger bicarbonate secretion, and inhibition of hydrogen secretion, which helps to lower the concentration of hydrogen ions delivered from the stomach. 

In stomach, UGN is produced by enterochromaffin-like cells. Those cells are major source of histamine after stimulation with gastrin and acetylcholine. Histamine regulates HCl production of the stomach [43]. The physiological and pathophysiological roles of UGN are the stomach are still not known; we can assume that UGN is involved in the protection of stomach from HCl by secreting bicarbonate in stomach lumen. Furthermore, GN is produced by parietal cells. After intravenous application of GN, those cells increase the production of protective mucus, which is one more protective mechanism in the stomach [56, 57].

In more details, when secreted in the gut lumen, GPs stimulate enterocytes via the membrane-bound guanylate cyclase C (GC-C) located at the apical membrane of the enterocytes. Activation of GC-C produces cGMP from GTP [7, 8]. The increase in cGMP as second messenger activates the protein kinase G II (PKG II) [58, 59] and inhibits the phosphodiesterase III (PDE III). Phosphodiesterases are divided into types concerning regulatory mechanism and substrate. PDE type III is responsible for the degradation of cAMP; therefore, inhibition of PDE III by cGMP leads to increase of intracellular concentration of cAMP which follows activation of cAMP-specific protein kinase A (PKA) [59–61]. Furthermore, cGMP partially inhibits Na^+^ absorption from intestinal lumen into the blood by inhibiting apical Na^+^/H^+^ exchanger type 2 (NHE2) and therefore preventing fast increase in blood sodium concentrations after the consumption of the salty meal [62, 63]. PKG II and PKA increase the secretion of Cl^−^ via the cystic fibrosis transmembrane conductance regulator (CFTR) followed by an activation of the member of Slc26 family which exchanges bicarbonate for chloride resulting in bicarbonate secretion. Two different opinions on how bicarbonate secretion occurs in intestine lumen as well as in other organs exist. Some researchers believe that bicarbonate is transported by CFTR itself. Since GPs still stimulate bicarbonate secretion in CFTR-deficient mice, it is reasonable to believe that the CFTR has a regulatory role while bicarbonate secretion is due to activation of members of Slc26 family [64]. Changes which follow GC-C activation in the intestine increase the amount of electrolyte in the intestinal lumen, which slows down the absorption of the water from intestinal lumen to the blood which could not be quickly excreted from the organism by the kidneys and protect from drastic increase in blood volume (Figure 4) [60, 65–67]. 

## 4. GC-C-Dependent Signaling Pathway

STa-specific binding sites are found in intestine and colon of mammals including human. Interestingly, binding sites are also found in extraintestinal tissue like gallbladder, trachea, testis, kidney, and opossum kidney cell line (OK cells) [68–70]. Since 1978, it is known that STa increased intracellular concentration of cGMP in intestine [24]; however, until 1990 and Schulz discovery the receptor responsible for STa effects in the gut was not known [23]. The discovered receptor was named guanylate cyclase C because it increases intracellular cGMP concentration and it is discovered after guanylate cyclase A (GC-A) and guanylate cyclase B (GC-B) receptors for atrial natriuretic peptides. Furthermore, CaCo-2 and T84 cell lines are intestinal human carcinoma cell lines. Intracellular concentration of cGMP increases in both cell lines after GPs stimulations via guanylate cycles C, and it is a well-established model for investigation of this main signaling pathway for GPs.

Main GPs receptor, GC-C, belongs to the family of guanylate cyclases. There are 2 major types of guanylate cyclases: soluble or cytoplasmatic guanylate cyclase which is receptor for NO and CO and membrane-bound guanylate cyclases which are receptors for natruretic peptides (Table 2). Soluble guanylate cyclases are widely spread (muscles, platelets, lung, liver, kidney, heart, and CNS) and recently well investigated as receptors of NO. Up to date, 6 membrane-bound guanylate cyclases are known. GC-A (also known as natriuretic peptide receptor type A, NPR-A) is receptor for atrial natriuretic peptide (ANP); it is a kidney isoform urodilatin and brain natriuretic peptide (BNP) and is involved in electrolyte and water homeostasis by heart. GC-A is also present in numerous tissue as well as soluble guanylate cyclase (smooth muscle, kidney, adrenal gland, heart, and CNS). GC-B or natriuretic peptide receptor type B, NPR-B, has a high affinity towards CNP. It is located at fibroblasts, heart, and CNS and plays more important role in the physiology of bones and cartilage. A splice variant of this receptor, NPR-Bi, is a truncated form expressed in human tissues, also binds CNP but lacks GC-function (it does not produce cGMP as second messenger but acts as a tyrosine kinase) [71, 72]. A fourth type of receptor, the natriuretic peptide receptors type C (NPR-C) is clearance receptor and binds all four natriuretic peptides. This receptor is G-protein-coupled receptor which is missing guanylate cyclase domain and guanylate cyclase activity. 

Guanylate cyclases D, E, and F (GC-D, GC-E, and GC-F) are located in sensory organs. Recently, GC-D was proposed to be a GPs receptor in olfactory system [73]. Guanylate cyclase G (GC-G or GC-1) is an orphan receptor present in the skeletal muscle, lung, intestine, testis, and kidney [74–76]. This orphan receptor could play important role in GPs signaling mechanism instead of GC-C in the kidney (see later). 

**Figure 5:**
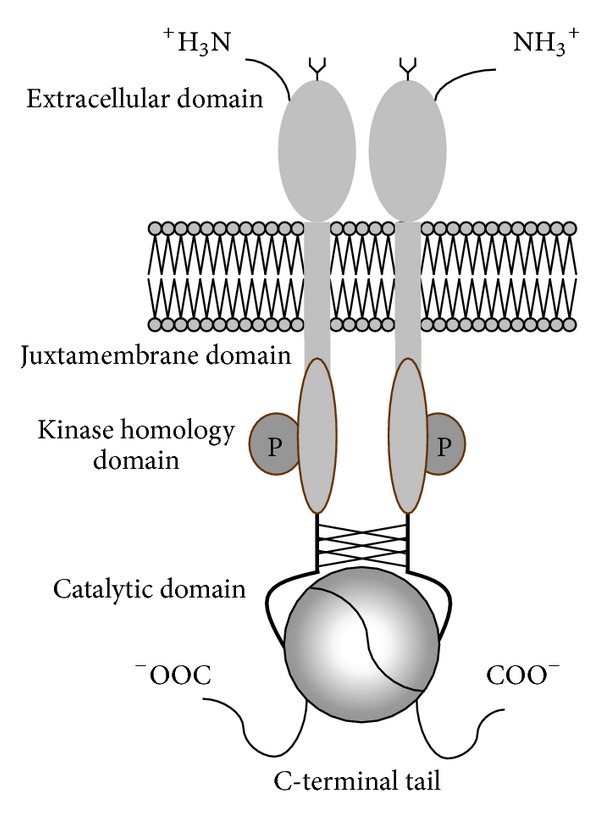
Guanylate cyclase C: extracellular domain is responsible for ligand binding. Juxtamembrane domain is located near the cell membrane and it is responsible for binding the G-proteins. Kinase homology domain needs to be phosphorylated for pronounced guanylate cyclase activity. Catalytic domain converts GTP to cGMP after ligand binding to extracellular domain. C-terminal tail of the GC-C is unique and it is involved in binding the GC-C to cytoskeleton.

GC-C is widely spread, localized in tissues expressing other parts of GP signaling pathway like CFTR (adrenal glands, brain, the embryonic or regenerating but not adult liver, placenta, testis, airways, spleen, thymus, and lymphatic nodes) [77–79]. GC-C expression is increased in infants and prematures which could explain higher sensitivity to presence of STa in intestine and more pronounced diarrhea in children [80]. Like other GCs, GC-C has extracellular, juxtamembrane domain, kinase homology, and catalytic domain (Figure 5). 

As any other transmembrane receptor, extracellular domain is responsible for ligand binding. Juxtamembrane domain is bound to extracellular domain by transmembrane domain and it is located near the cell membrane. This domain has similar structure to parts of IGF (insulin-like growth factor) and EGF (epidermal growth factor) receptors which are responsible for binding the G-proteins [81, 82]. Kinase homology domain is similar to catalytic domain of PDGF (platelet-derived growth factor) receptors. Catalytic domain converts GTP to cGMP after ligand binding to extracellular domain. For pronounced guanylate cyclase activity of GC-C kinase, homology domain should be phosphorylated. C-terminal tail of the GC-C is unique and it is involved in binding the GC-C to cytoskeleton and important for endocytosis of ligand-receptor complexes [83].

Protein kinase C (PKC) is a well-established regulator of GC-C activity. PKC phosphorylates C-terminal tail of GC-C which leads to 70% increase in intracellular concentration of cGMP after STa stimulation compared to control [84–88]. For the full activity of GC-C, Mg^2+^-ATP should be present [89].

## 5. GC-C-Independent Signaling Pathway

The evidence of the presence of additional receptors and signaling pathways for GPs exist and it is found mostly in the kidney but also in other tissues like intestine. Further research should be done to determine physiological and pathophysiological roles of all GPs signaling pathways in all organic systems that are expressing GPs. The first evidence of existing a GC-C-independent signaling pathway was given in intestine where was shown that localization of GC-C mRNA and binding sites for STa and GPs in the intestine are not identical [90]. Since STa is a more potent activator of GC-C, it is reasonable to believe that it could activate all GPs receptors which is later shown in the kidneys. 

Two populations of binding sites for STa in the intestine have been identified: high affinity receptors not coupled to the guanylate cyclase (only 5% of binding sites) and low affinity binding sites coupled to the guanylate cyclase (95% of binding sites) [91]. Those 2 types of binding sites could occur because of regulation of activity of GC-C itself or could present two different receptor types for GPs. Furthermore, GC-C-independent binding sites are located at the basolateral membrane of colonocytes [92], and those receptors and signaling pathways are still not identified. GC-C-deficient mice are resistant to intestinal secretion produced by STa [93–95]; however, 10% of the STa intestinal binding sites are still present possibly involving Ca^2+^ signaling pathway with activation of PKC [96, 97].

Necessity of further research for GC-C-independent signaling pathway for GP became more evident when renal effects were still observed in GC-C-deficient mice [5]. GN, UGN, and STa still change membrane potential via changes in membrane conductances of principal cells in isolated cortical collecting ducts of GC-C-deficient mice [99]. G-protein coupled receptor which activates phospholipase A_2_ was suggested to be a part of cGMP-independent signaling pathway in the mouse and human kidney [98, 99]. After activation of this receptor, intracellular concentration of arachidonic acid increased and changed cell conductances for ions which could lead to natriuresis, kaliuresis, and diureis present in GC-C-deficient mice. Additional signaling pathway is investigated in more detail in different parts of mouse and human kidney nephron segments (see later). Furthermore, GC-C knock-out animals have normal blood pressure. If the GC-C is the only receptor for GPs, UGN-deficient mice should have the normal blood pressure as well which is not the case. Surprisingly, UGN-deficient mice are developing hypertension [100, 101], suggesting the effects of UGN on blood pressure via GC-C-independent signaling pathway. Further research should be done to investigate the importance of GC-C-independent signaling pathway in physiology and pathophysiology in extrarenal tissues and blood pressure regulation.

## 6. Signaling Pathways of Guanylin Peptides in the Kidney

The main physiological function of GP is to increase salt excretion via kidneys after increased per os intake. However, the effects of the GN and UGN on the kidney nephron segments are slightly different assuming more important physiological role of UGN in maintaining sodium homeostasis. Humans consuming the diet with high amount of salt (10 g/day) excrete more UGN in the urine compared to people at low-salt diet (7 g/day) [29]. In rats on high-salt diet, also increased excretion of UGN as well as cGMP in the urine compared to normally fed animals, suggesting involvement of cGMP signaling pathway in UGN action in the kidney [102]. Furthermore, in UGN-deficient mice natriuresis produced by oral salt load is decreased [100].

GN has mostly kaliuretic effects in the kidneys; therefore, it was reasonable to believe that GN could play a significant role in potassium secretion after increased dietary potassium intake. Recently, Oh et al. showed that GN and UGN are not involved in intestinal sensing of dietary potassium intake and still unknown hormones stimulate kidney potassium excretion due to increased potassium intake [103]. As can be expected by structure containing 3 disulfide bonds (Figure 1), STa effects in the kidneys are more pronounced compared to GPs as it is shown for intestine obviously activating all signaling pathways present [21, 61].

The action of the GPs could be produced by the intestine with distant endocrine effects on the kidneys and/or paracrine produced locally by the kidney itself. Recent research implicates paracrine effects of GP in the kidney. UGN but not GN kidney expression increases after increased oral salt load. Paracrine production of GP in the kidney is due to still unknown linkage mechanism between intestine and kidney. Kuhn's laboratory suggested that renal UGN expression is influenced by renal hypertonicity but not by intestinal sensing of salt intake. Cultured murine M-1 cells, cells with properties of cortical collecting duct cells, are showing UGN but not GN expression. UGN expression increases when M-1 cells were put in hypertonic conditions (using hypertonic NaCl solution or mannitol). Further investigations should be done to clarify how increased intestinal sodium load increases UGN expression in the kidney [20].

**Figure 6:**
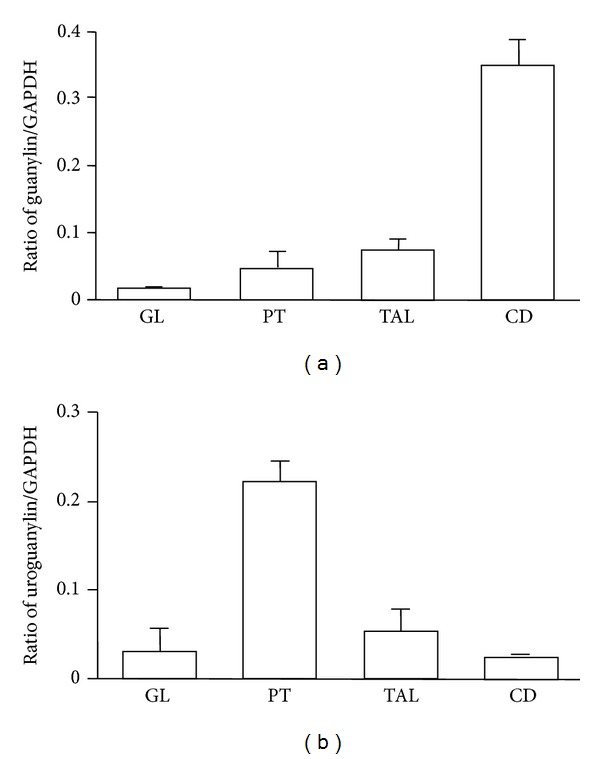
Relative mRNA expression of (a) guanylin (GN) and (b) uroguanylin (UGN) in different mouse nephron segments. GL: glomerulus, PT: proximal tubule, TAL: thick ascending limb of Henle's loop, CD: collecting duct, and GAPDH: glyceraldehyde-3-phosphate dehydrogenase. Mean values: SEM, *n* = 4. Modified from Potthast et al. [20].

Similar to the difference in the axial expression along the intestine (Figure 2), GN and UGN are not expressed equally along the different nephron segments suggesting the similarity in paracrine function between intestine and kidney. mRNA for GN is more expressed in the collecting duct (distal nephron segments), in analogy to GN expression in the colon while mRNA for UGN is present mostly in the proximal tubule, again comparable to the higher expression in proximal intestine (Figure 6) [20]. Goy's laboratory recently found pro-UGN (the uroguanylin precursor) in distal nephron segments with more pronounced natriuretic effects compared to UGN itself, but they failed to show expression of GC-C in the kidney [104, 105].

Expression of GC-C in the kidney is still controversial. Like in intestine, localization of mRNA for GC-C differs from binding site for STa in the kidney. Potthast et al. showed the expression of GC-C in glomeruli and proximal tubules but not in Henle's loop and cortical collecting ducts [20, 21, 98]. Exact localization of GC-C is still not known because Carrithers et al. localized GC-C in all parts of rat nephron while Goy's laboratory failed to show the expression of GC-C in the kidney [105, 106]. From other members of GC-C signaling pathway of GP, PKG II is also expressed in the kidneys. mRNA for PKG II is located in all nephron segments with the highest expression in glomeruli and proximal tubules where the GC-C is also expressed the most [20, 106]. However, the involvement of cGMP and GC-C in the signaling pathway of GPs in proximal tubule cells is obviously not present in the effects of GPs described in GC-C-deficient mice, and GC-C-independent signaling pathway in the kidneys was discovered.

Even the GPs are expressed in different parts of the nephron segments, the most effects of GP are mostly investigated in proximal tubules and collecting duct. Since GPs increase the secretion of sodium, potassium, and water in the kidney without changes in glomerular filtration rate or renal blood flow, the effects of GPs in glomeruli are still unknown. 

## 7. Signaling Pathways of Guanylin Peptides in the Proximal Tubules

The first discovered localization of GPs action is proximal tubule. Proximal tubule cells are specific for their massive ion and water transport. Each day proximal tubules reabsorb 120 L of filtrated water, 65% filtrated sodium, potassium and chloride, and almost all filtrated bicarbonate. Small changes in proximal tubule transport will lead to massive changes in final urine.

Binding sites for GN, UGN, and STa are located at opossum and human proximal tubule cells [69, 70, 107, 108]. In rat proximal tubules, binding sites for STa are found at apical and basolateral cell membranes as well as in basolateral cell membrane of Henle's loop and medullar collecting duct cells [109]. The possible physiological role for receptors for GPs located at basolateral side of kidney nephron segments is not clear yet. GPs increase intracellular cGMP concentration in a proximal tubule cell line of opossum kidney (OK cells), in kangaroo proximal tubule cells (PtK-2 cells), and in an immortalized human proximal tubule cell line (IHKE1) [21, 68, 77, 110]. 

Proximal tubule is responsible for more than 90% of filtrated bicarbonate reabsorption. For each hydrogen ion transported via apical membrane, one bicarbonate is reabsorbed back into peritubular capillaries. UGN inhibits the activity of Na^+^/H^+^ exchanger isoform 3 (NHE3) located at the apical membrane which decreases the secretion of hydrogen ions and therefore decreases the reabsorption of bicarbonate. UGN inhibits NHE3 activity via cGMP followed by PKG activation and cAMP followed by PKA activation, both of which phosphorylate NHE3 already located at apical membrane of the cells. UGN also reduces NHE3 surface expression [111]. Recently discovered renoguanylin (eel isoform: A D L C E I C A F A A C T G C L) also inhibits hydrogen transport at the apical membrane of proximal tubules in the rat kidneys. Renoguanylin inhibits NHE as well as H^+^-ATPase and is therefore involved in the regulation of hydrogen and bicarbonate transport as other guanylin peptides [112]. Human isoform of renoguanylin is not discovered yet.

**Figure 7:**
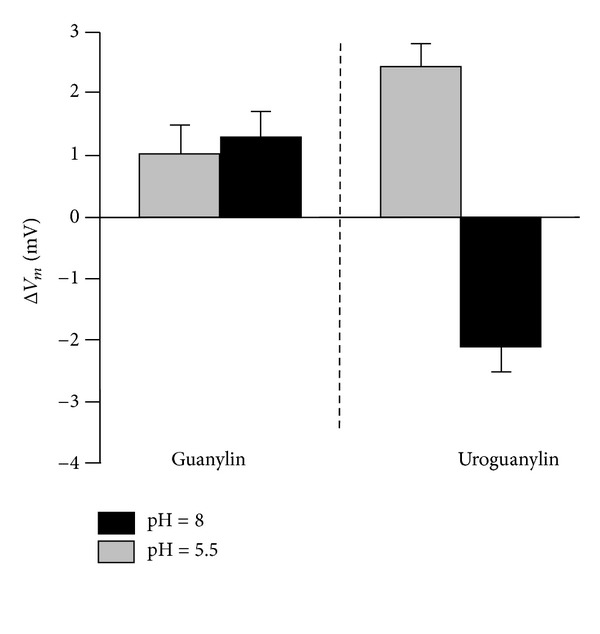
pH dependence of effects of guanylin peptides on membrane voltages (*V*_*m*_) of human proximal tubule cells. Guanylin (GN) depolarized cells at pH 5.5 as well as pH 8.0. Uroguanylin (UGN) at pH 5.5, activated GC-C and depolarized cells while at pH 8.0 decreased affinity of GC-C for UGN and GC-C independent, G-protein-coupled receptor is activated which leads to hyperpolarization of the cells. Mean values: SEM. Modified from Sindiće et al. [21].

GP regulate not only transport of hydrogen and bicarbonate in proximal tubules, as shown previusly, also change sodium and potassium conductances and electrogenic electrolyte transport in IHKE-1 cells which are a human proximal tubule cell line. GPs are reaching the receptors located at apical membrane of those cells. GPs show different effects in the kidney, UGN mostly causing natriuresis while GN kaliuresis; it was reasonable to expect that the effects on those hormones on proximal tubule cell line differ as well. GN mostly activates cGMP-dependent signaling pathway. Since GPs increase excretion in the urine as well as cGMP due to high-salt diet which is in contrast to still existing effects of GPs in GC-C-deficient mice, another guanylate cyclase could play a role as receptor for GPs in proximal tubules. Guanylate cyclase present in opossum kidney (OK-GC) has a 92–95% identity in the catalytic domain but only 55–58% identity in ligand-binding domain compared with rat, pig, and human GC-C. Human isoform of this still unidentified receptor could play a possible role in cGMP-dependent but GC-C-independent signaling pathway of GPs [110]. In IHKE-1 cells, UGN and STa activate both cGMP-dependent and cGMP independent signaling pathway. UGN also activates both signaling pathway in pig proximal tubule cell line (LLC-PK1). Which signaling pathway will be activated depends on concentrations of the hormone and pH. As it was shown for GC-C in intestine (Figure 3), in proximal tubule cells UGN activates GC-C and depolarized cells (as well as cGMP) at pH 5.5. When the pH values change to 8.0, we can assume that affinity of GC-C for UGN decrease and other GC-C-independent signaling pathway could be more pronounced which leads to UGN-dependent hyperpolarization of the cells (Figure 7). Could this switch in signaling pathways due to pH differance play any physiological role in regulation of sodium, hydrogen, bicarbonate transport versus potassium transport needs to be established.

Preincubation of IHKE-1 cells with pertussis toxin (PT), an inhibitor of G-protein coupled receptors, has no effect on depolarizations neither on cGMP accumulation caused by GPs. However, hyperpolarization caused by UGN is inhibited by PT suggesting that the second receptor activated by UGN and probably also by STa is a PT-sensitive G-protein coupled receptor [21].

## 8. Signaling Pathways of Guanylin Peptides in the Collecting Ducts

Final hormonal regulation of renal electrolyte and water homeostasis takes place in the collecting duct (CD). They have at least 2 types of the cells: principal cells which are responsible for K^+^ secretion (ROMK channels), Na^+^, and water reabsorption (ENaC and aquaporins 2, 3, and 4, resp.) and intercalated cells responsible for K^+^ reabsorption via ATPases. We already discussed the importance of sodium regulation, but regulation of potassium plasma concentration is even more important because of its serious consequences on heart function in hyperkalemic conditions. There are numerous mechanisms for potassium homeostasis regulation by the kidney from aldosterone to direct regulation by plasmatic potassium concentration. Due to aldosterone activation of Na^+^/K^+^ ATPase and increased apical membrane conductances for sodium (ENaC) and potassium (ROMK) of principal cells, sodium is reabsorbed in the blood and potassium is secreted in the tubular lumen. To be able to reabsorb potassium, intercalated cells have H^+^/K^+^-ATPase at the apical membrane which secrete hydrogen ions and reabsorb potassium. Since GPs at the same time induce natriuresis and kaliuresis, it is reasonable to believe that those hormones regulate water and electrolyte transport in principal cells as well as in intercalated cell of collecting ducts. Since GN showed more pronounced kaliuresis, its physiological importance in potassium homeostasis in contrast to aldosterone is suggested and future research should clarify physiological importance of GN in the kidney.

Collecting ducts are cortical collecting duct (CCD) and medullary collecting duct (MCD). Even the final regulation of electrolyte and especially water transport is located at the end part of the nephron, MCDs; however, most of the research of GPs signaling pathways in the kidney nephron segments was done at CCDs.

### 8.1. Signaling Pathways of Guanylin Peptides in Principal Cells of CCD

**Figure 8:**
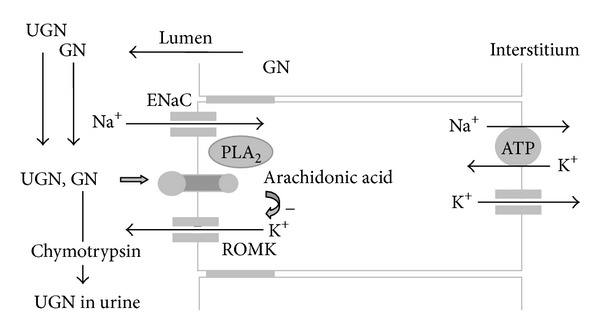
Signaling pathways of guanylin (GN) and uroguanylin (UGN) in the principal cell of the cortical collecting ducts. GN present from glomerular filtration or from paracrine secretion via proximal parts of the nephron segments is degradated by peptidase and therefore not present in the urine. Guanylin peptides (GN and UGN) activate phospholipase A_2_ (PLA_2_), which leads to an increase in the intracellular concentration of arachidonic acid and an inhibition of luminal ROMK K^+^ channels. ENaC: epithelial sodium channels.

GPs change membrane conductances and therefore membrane voltages of principal cells of wild-type mice and human CCD. The same results are presented for principal cells isolated from CCDs of GC-C-deficient mice which are in line with the observation that GC-C-deficient mice still exert natriuresis, kaliuresis, and diuresis upon GPs infusion [5, 98, 99]. The source of UGN reaching the lumen of the collecting duct is filtrated from the blood where secreted by intestine and/or from secretion by proximal tubule cells [20]. It looks like the GN present in lumen of CDs could be secreted only by local cells since GN is degradated with chymotrypsin which is located in all parts of the nephron, and mRNA for GN is most present in the cells of collecting ducts. Since UGN, in contrast to GN, is resistant to degradation by luminal proteases and probably secreted by proximal tubule where the expression of mRNA for UGN is the highest, UGN will be concentrated along the nephron due to volume reabsorption and will be present in the final urine (Figure 8) [33].

It is already established that GN and UGN have different effects on the kidney in *in vivo* experiments. In mouse and human principal cells of cortical collecting ducts, they express slightly different effects which are species specific while differ in mouse and human principal cells.

In mouse principal cells of CCDs, GPs activate receptors located at apical membrane similar to effects of GPs in the intestine. GN mainly depolarized cells while UGN, STa, and membrane permeable cGMP (8 Br cGMP) hyperpolarized cells due to changes in potassium conductances (depolarizations due to a decrease, hyperpolarizations due to an increase in a K^+^ conductance) suggesting different sensitivities of the two signaling pathways for GPs. However, hyperpolarizations caused by GPs (activation of cGMP-dependent signaling pathway) are still present in principal cells from CCDs isolated from GC-C-deficient mice. In the same guanylate cyclase C-deficient mice, GPs still increase the cGMP concentration in urine, indicating an involvement of another guanylate cyclase than guanylate cyclase C [5]. GC-G is other type of guanylate cyclase present in mouse collecting duct which could be responsible for cGMP- and PKG-dependent signaling pathways of GPs in those cells. It is known that PKG activates Ca^2+^-dependent K^+^ channels which could be responsible for PKG-dependent hyperpolarizations caused by GPs. cGMP-independent signaling pathway (depolarizations caused by GPs) involves G-coupled receptor (could be the same as already shown in proximal tubule cell line) which activates phospholipase A_2_ and release of arachidonic acid. Since arachidonic acid inhibits potassium channels, ROMK, located at apical membrane of principal cells, those channels could be the target protein of cGMP-independent GPs action in principal cells [99, 113]. 

Recently, the first electrophysiology study of principal cells of human CCDs was performed. Starting membrane potential of human principal cells is not different from starting membrane potential of mouse and rat principal cells which assumes similar potassium conductances between different species. However, only one signaling pathway for GPs is present in human principal cells. GPs depolarized while again membrane permeable analog of cGMP hyperpolarized cells clearly identify signaling pathway for GPs as GC-C and cGMP independent. Ca^2+^ and PKC are suggested as a possible additional and cGMP-independent signaling pathway for GPs in the intestine but that was not the case for principal cells because the GPs are not changing intracellular concentration of calcium. As it is shown for mouse, depolarizations of human principal cells caused by GPs are due to inhibition of potassium conductances, which consequently leads to reduction of driving force for sodium reabsorption and therefore possible natriuresis. The remaining question is if GPs could inhibit potassium reabsorption in intercalated cells and therefore induce kaliuresis as well. However, ROMK-deficient mice show natriuresis, kaliuresis, and diuresis, a pattern of responses so similar to mice stimulated with GPs which is still not fully understood [114]. As it was shown for human principal cells as well as for mouse principal cells, cGMP-independent signaling pathway for GPs actually leads to inhibition of ROMK channels by arachidonic acid therefore, the effects of GPs on kidneys could share the same mechanisms of natriuresis, kaliuresis, and diuresis as is present in ROMK-deficient mice [98].

Activation of G-protein coupled receptor which leads to activation of PLA_2_ and increase of intracellular concentration of arachidonic acid is shown by G-protein coupled receptor 14 (GPR14) [115]. Two types of the cells, human embryonic cell line (HEK283) cells and Chinese hamster ovary (CHO) cells overexpressing GPR14 receptor, showed different effects of GPs. Only HEK293 cells obviously express all necessary proteins which makes them suitable for this kind of studies in contrast to CHO cells suggesting the importance of expression system in investigating possible receptors for GPs. Depolarization of HEK293-GPR14 cells induced by GPs is higher compared to WT cells [116]. Whether this is the G-protein coupled receptor activated by GPs actions in the different mouse and human nephron segments needs to be further investigated.

### 8.2. Signaling Pathways of Guanylin Peptides in Intercalated Cells of CCD

It is far less known about signaling pathway of GPs in the intercalated cells. Resent work from Zelinkovic's laboratory suggested the effects of UGN on Cl^−^/HCO_3_^−^ exchanger, pendrin, located in intercalated cells [55]. In apical membrane of intercalated cells type non-A, non-B of CCD, pendrin, functions as Cl^−^/HCO_3_^−^ exchanger where it is involved in bicarbonate transport. Pendrin is proposed to be involved in the functions of aldosterone and angiotensin II. Even UGN originally works opposite to those hormones, it could regulate the expression of this anion exchanger. Physiological function of this regulation needs more clarification.

### 8.3. Signaling Pathways of Guanylin Peptides in MCD

It is less known about the function of GPs in the last part of the nephron, medullary CD. GPs decrease the cell volume and increase the luminal space which suggests secretion of water and consequently diuresis [109]; however, GPs do not change water permeability in inner medullary CD cells [117].

## 9. Cellular Effects of Guanylin Peptides in Other Organs

Patients with cystic fibrosis have pathological changes in water and electrolyte transport in the pancreas, the liver, the lung, and the intestine. It might be speculated that GPs are also involved in the physiology of those organs.

GPs and parts of their signaling pathway are present in the pancreas. mRNA for GC-C and UGN are detected in the cells of the exocrine part of the pancreas, and GN increases intracellular cGMP *via *GC-C in a human pancreatic cell line [118–120]. In pancreas, GPs could be involved in a very demanding bicarbonate secretion necessary for acidity neutralization in duodenum.

In the liver, GN is located in the epithelial cells of bile ducts and of the gallbladder. Other members of GC-C signaling pathways of GPs (GC-C, PKG II, CFTR, and anion exchanger Slc4a2) are located at the apical membrane of the same cells suggesting physiological role of GN in production of hepatic and cystic bile via regulation of water and electrolyte transport by paracrine signaling pathway [121]. mRNA for GC-C is present in fetuses and newborn rats, after partial hepatectomies, and during liver regeneration suggesting an additional role of GPs in those conditions [79, 94, 122, 123].

Lungs are one of the most damaged organs in cystic fibrosis. In healthy individuals, GN is secreted by airways where it activates a well-known GC-C signaling pathway and leads to CFTR activation [70, 78, 79, 124, 125]. Furthermore, GPs relax tracheal smooth muscle cells and mucus production in large and small airways, which suggests UGN as a new treatment of asthma [126, 127].

GPs and parts of the GC-C-dependent signaling pathway are also found in sweat glands, the adrenal medulla, and the male reproductive system [128–130]. Recently it was discovered that UGN relaxes human corpus cavernosus and could be used in the treatment of erectile dysfunction [131]. Even the appetite is proposed to be regulated by circulatory pro-UGN secreted from the intestine and binding to GC-C in hypothalamus [132].

According to this limited information on the effects of GPs on various organs, the GC-C-dependent signaling pathway seems to be the predominant signaling pathway in most organs and only in the kidney does the signaling involve different receptors and signaling mechanisms. Like it is shown for the regulation of blood pressure by UGH, further studies are necessary to determine the possible physiological function of GC-C-independent signaling pathways of GPs in extrarenal tissues.

## 10. Guanylin Peptides in Pathophysiology

The most investigated pathological conditions which are involving GPs are intestinal tumors. GPs are less expressed in colon adenocarcinoma, adenoma, and intestine polyps in mouse and human compared to healthy individuals suggesting involvement of GPs in the development of intestinal tumors [133–135]. Indeed, GPs regulate proliferation and differentiation, prolong the cell cycle, and induce apoptosis of T84, CaCo-2 cells, and mouse intestinal cells, and oral application of UGN leads to a decrease in the number and size of polyps in mice that develop intestinal polyposis [133, 136–140]. Like UGN, nonsteroidal antiinflammatory drug as well present their anticancer effects via increase in cellular levels of cGMP [135, 141, 142]. Among other factors, like differences in type and food processing, increased levels of cGMP induced by frequent diarrhea in third world countries are presumed to be protective against intestine cancers. Furthermore, ectopic expression of GC-C could be used as marker for development of metastasis in lymphatic system of esophageal, stomach, and colon cancer [143–146].

Cystic fibrosis (CF) is a genetic disease caused by mutations of cystic fibrosis transmembrane conductance regulator (CFTR) mostly affecting lungs, pancreas, liver, and intestine. CFTR is chloride channel which regulates membrane conductances of the affected cells for, as far as we know, chloride, sodium, and bicarbonate; reduced amount of electrolytes in lumen results in less water secretion and production of thick mucus which blocks airways in the lung, pancreatic ducts, and bile ducts in the liver and causes meconium ileus. This intestinal obstruction in the newborn could be seen in CF patients, is connected to mutations of the gene for guanylate cyclase C [147]. Furthermore, GPs still stimulate bicarbonate secretion in CFTR-deficient mice, and recently Slc26a4 (pendrin) was suggested as target for UGN-dependent chloride transport in intercalated cells of kidney cortical collecting duct suggesting the existence of CFTR-independent signaling pathway for GPs in cystic fibrosis patients [64]. How GPs help dilute thick mucous secretion in the intestine via GC-C-dependent signaling but CFTR-independent signaling pathway needs to be established. Although life span of patients suffering from CF is significantly increased, the therapy is still symptomatic. Since the GPs colocalize with CFTR, future research should be done to determine whether GPs regulation of water and electrolyte transport has any physiological effect on the lung, pancreas, and intestine of CF patients (via GC-C-independent signaling pathways) and could GPs effects on ion transport be used as a new therapy approach in patients with cystic fibrosis. 

GPs are also involved in different kidney diseases. Concentrations in plasma GPs increase in patients with chronic renal failure and glomerulonephritis and in patients on hemodialysis [27, 29, 30, 40, 148]. In patients with nephritic syndrome, UGN plasma concentration was higher and urinary concentration was lower compared with values in healthy volunteers possibly because kidney is damaged and it has reduced capability to metabolize and excrete GPs [40]. Recently, in experimental nephrotic syndrome, changes in UGN concentrations in urine and plasma corresponded to changes in Na^+^ excretion; therefore, UGN could be used as natriuretic peptide in nephritic syndrome [149, 150]. 

## 11. Conclusion 

GPs are well known as intestinal natriuretic peptides with well-established function in the intestine and the kidneys. GN and UGN regulate electrolyte and water transport in the intestine and cause kaliuresis, natriuresis, and diuresis in the kidney. After a salty meal, GPs decrease sodium reabsorption from the intestine and induce sodium secretion by the kidneys and therefore prevent postprandial hypernatraemia which could occur.

The main signaling pathway for GPs includes GC-C and increase intracellular concentration of cGMP followed by activation of PKG II, inhibition of Na^+^/H^+^ exchange, and CFTR activation. Additional GC-C-independent signaling pathway exists in intestine but is still not well understood; however, this signaling pathway is more investigated in the kidney, and it is suggested to be more important for GPs action in the kidney than GC-C-dependent signaling pathway. It is established that other signaling pathways involve G-coupled receptors (probably GPR14), the molecular identity receptors for GPs in the kidney and other extrarenal tissues remain to be determined. 

We have known that GPs and their signaling pathways exist in other organs like liver, pancreas, lung, sweat glands, and male reproductive system and they are involved in different pathological conditions like cystic fibrosis, asthma, intestinal tumors, and kidney and heart failure. Future research should investigate the importance of GC-C-independent signaling pathways in extrarenal tissues and determine new treatments for pathological conditions.

## Figures and Tables

**Table 1:** Sequences of guanylin and uroguanylin in different species.

Guanylin	
Eel	Y D E **C E I C** M F **A A C** T **G C**
Opossum	S H T **C E I C** A F **A A C** A **G C**
Human	P G T **C E I C** A Y **A A C** T **G C**
Rat/mouse	P N T **C E I C** A Y **A A C** T **G C**
Pig	P S T **C E I C** A Y **A A C** A **G C**
Guinea pig	P S T **C E I C** A Y **A A C** A **G C**

Uroguanylin	
Eel	P D P **C E** I **C** A **N** A **A C T G C** L
Opossum	Q E D **C E** L **C** I **N** V **A C T G C**
Human	N D D **C E** L **C** V **N** V **A C T G C** L
Rat/mouse	T D E **C E** L **C** I **N** V **A C T G C**
Pig	G D D **C E** L **C** V **N** V **A C T G C** S
Guinea pig	N D E **C E** L **C** V **N** I **A C T G C**

Identical amino acids in these proteins of different species are indicated in bold.

**Table 2:** Expression of cytoplasmic and membrane-bound guanylate cyclases.

Cyclases	Tissue distribution	Agonists
Cytoplasmic	Skeletal muscle, platelets, lung, liver, kidney, heart, and CNS	NO, CO
GC-A	Smooth muscle, kidney, adrenal gland, heart, and CNS	ANP, BNP
GC-B	Fibroblasts, heart, and CNS	CNP
GC-C	Intestine, adrenal gland, CNS, lung, reproductive glands, and kidney	STa, GN, and UGN
GC-D	Olfactory cells	GN, UGN
GC-E	Retina	Unknown
GC-F	Retina	Unknown
GC-G	Skeletal muscle, lung, intestine, and kidney	Unknown

ANP: atrial natriuretic peptide, BNP: brain natriuretic peptide, CNP: C-type natriuretic peptide, CNS: central nervous system, CO: carbon monoxide, NO: nitric oxide, STa: heat-stable enterotoxin of *Escherichia coli*, GN: guanylin, and UGN: uroguanylin.
